# Supplementary material for: Atomic Scale Optimization Strategy of Al-Based Layered Double Hydroxide for Alkali Stability and Supercapacitors
Source: Int J Mol Sci. 2022 Oct 1;23(19):11645. doi: 10.3390/ijms231911645 (PMC9569664; doi:10.3390/ijms231911645)
Supplement: Supplementary file 1 [file ijms-23-11645-s001.zip › ijms-1907573-supplementary.pdf]

Supplementary Information

Figure S1. XRD patterns of NiAl LDH at different concentrations of KOH.

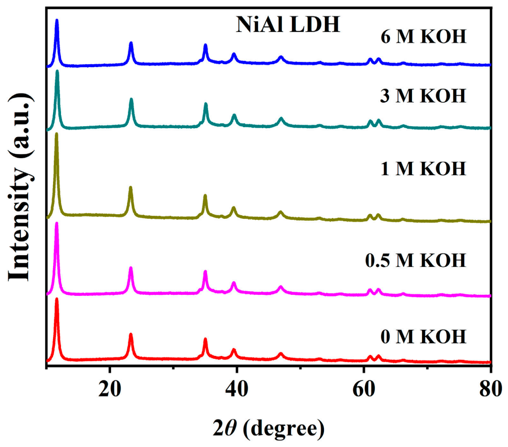

Figure S2. (a) XRD patterns of CoAl LDH at different concentrations of KOH, (b) partial enlarged drawing of (a).

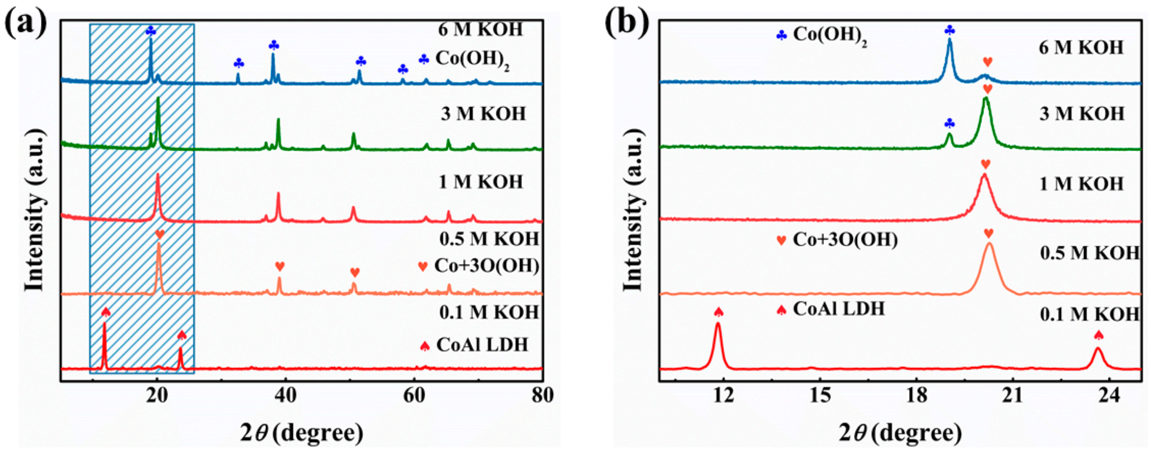

Figure S3. (a) CV, GCD curves and rate capacities of NiAl LDH, CoAl LDH and CoAl LDH-E.

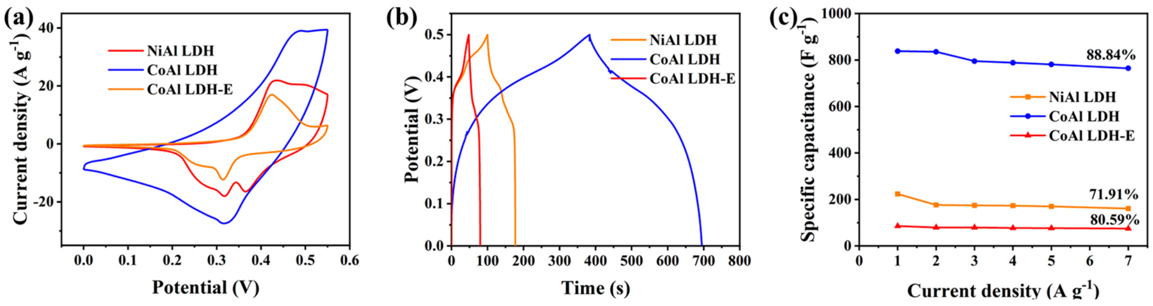

Figure S4. (a) The morphology, (b) selected area electron diffraction, (c) elements content and (d) EDS mapping of Ni_2Co_1Al LDH

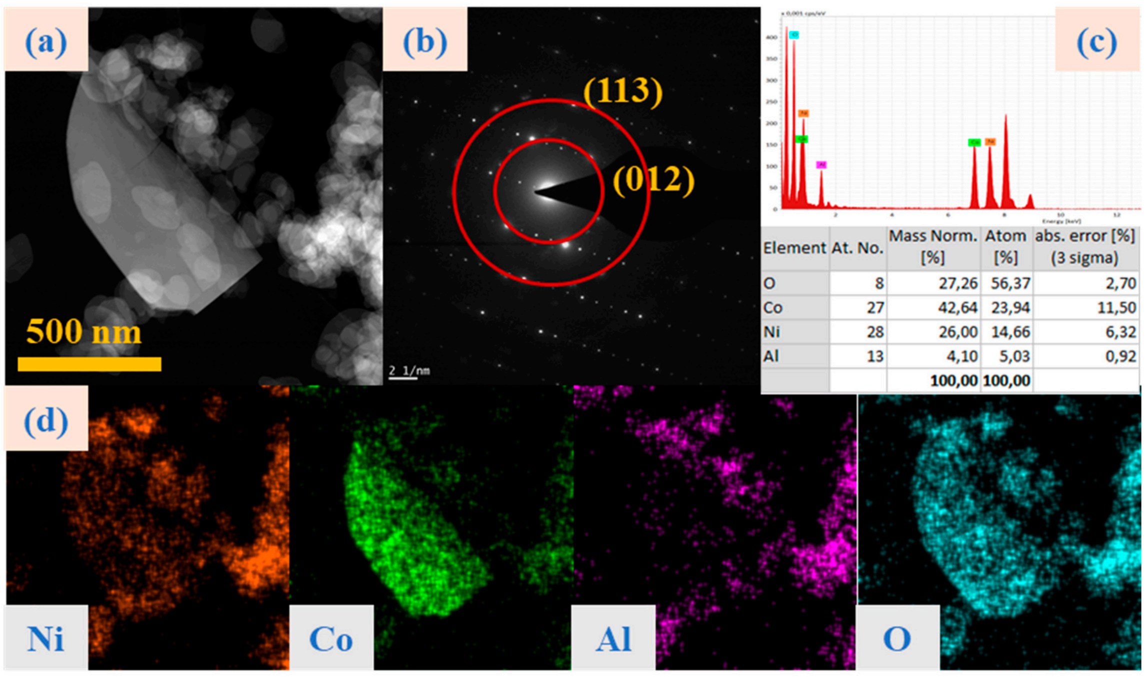

Element	At. No.	Mass Norm. [%]	Atom [%]	abs. error [%] (3 sigma)
O	8	27,26	56,37	2,70
Co	27	42,64	23,94	11,50
Ni	28	26,00	14,66	6,32
Al	13	4,10	5,03	0,92
		**100,00**	**100,00**	

Figure S5. (a) The morphology, (b) selected area electron diffraction, (c) elements content and (d) EDS mapping of Ni_2Co_1Al LDH-E.

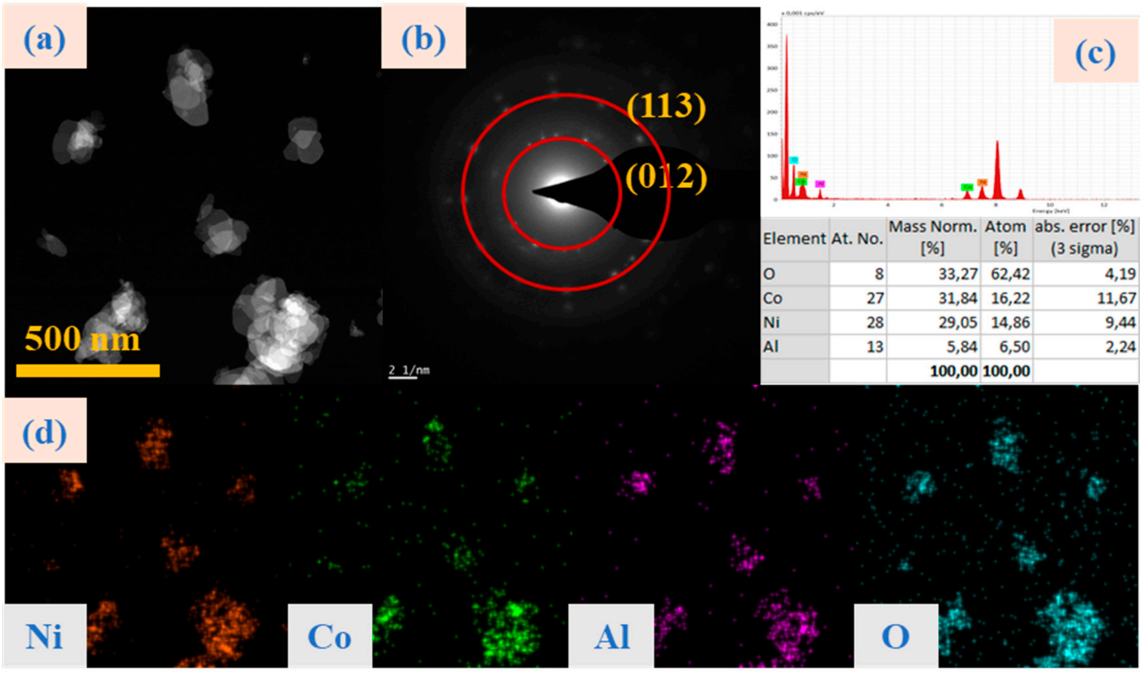

Element	At. No.	Mass Norm. [%]	Atom [%]	abs. error [%] (3 sigma)
O	8	33,27	62,42	4,19
Co	27	31,84	16,22	11,67
Ni	28	29,05	14,86	9,44
Al	13	5,84	6,50	2,24
		**100,00**	**100,00**	

Figure S6. The CV, GCD curves and Nyquist plots of Ni_2Co_1Al LDH-E, Ni_1Co_2Al LDH-E, $Ni_2Co_1S_4$-E and $Ni_1Co_2S_4$-E.

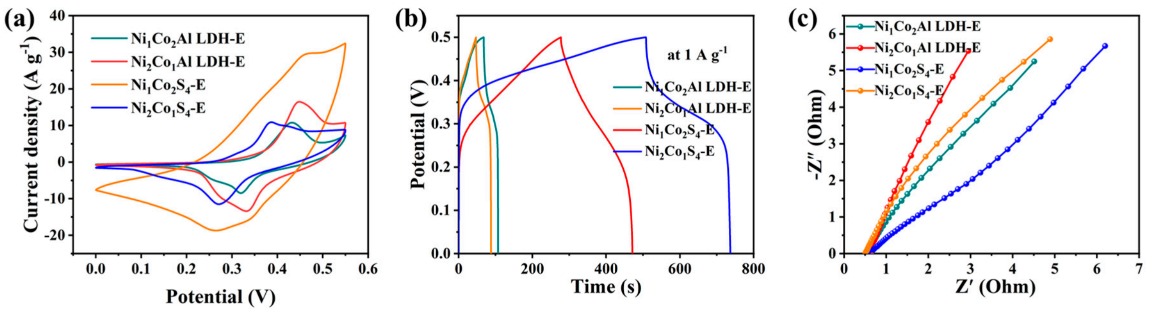

Figure S7. The GCD curves of AC at the current densities of 1, 2 and 3 A g^{-1}.

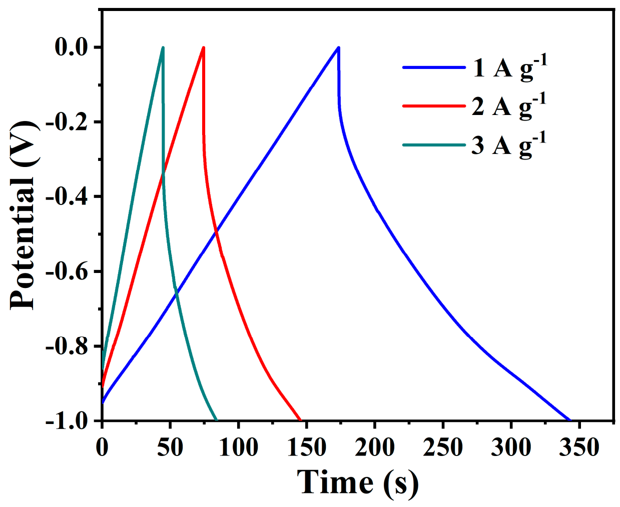

Table S1. The specific surface area, pore volume and average pore diameter of $Ni_2Co_1S_4$, $Ni_2Co_1S_4$-E, Ni_2Co_1Al LDH and Ni_2Co_1Al LDH-E.

	$Ni_2Co_1S_4$	$Ni_2Co_1S_4$-E	Ni_2Co_1Al LDH	Ni_2Co_1Al LDH-E
Specific surface area （m^2 g^{-1}）	7.1648	31.0860	58.8592	63.2865
Pore volume （cm^3 g^{-1}）	0.040893	0.151196	0.376361	0.372464
Average pore diameter （nm）	18.4100	14.9734	13.6747	13.0023
